# Correction: Turn-on fluorescent glucose transport bioprobe enables wash-free real-time monitoring of glucose uptake activity in live cells and small organisms

**DOI:** 10.1039/d5cb90030a

**Published:** 2025-07-03

**Authors:** Monica S. Hensley, David Hutchings, Aldelrahman Ismail, Micaela Rayne Geborkoff, Thomas Werner, Marina Tanasova

**Affiliations:** a Department of Chemistry, Michigan Technological University 1400 Townsend Dr Houghton MI 49931 USA mtanasov@mtu.edu; b Health Research Institute, Michigan Technological University 1400 Townsend Dr Houghton MI 49931 USA; c Department of Biological Sciences, Michigan Technological University 1400 Townsend Dr Houghton MI 49931 USA

## Abstract

Correction for “Turn-on fluorescent glucose transport bioprobe enables wash-free real-time monitoring of glucose uptake activity in live cells and small organisms” by Monica S. Hensley *et al.*, *RSC Chem. Biol.*, 2025, **6**, 987–995, https://doi.org/10.1039/D4CB00239C.

The authors regret that two contributors, Micaela Rayne Geborkoff and Professor Dr Thomas Werner, were inadvertently omitted from the original author list of this article. Both individuals made significant contributions to the research reported.

Micaela Rayne Geborkoff was involved in the design, planning, and execution of the *Drosophila* experiments, specifically in the growth and treatment of fruit fly larvae. Professor Dr Thomas Werner provided laboratory resources and supervision, and played a key role in the experimental design and execution of the *in vivo* work, particularly contributing to the results presented in Fig. 7.

These contributions meet the journal's authorship criteria, and the corrected author list is as follows:

**Monica S. Hensley,** ^**a**^ **David Hutchings,** ^**a**^ **Aldelrahman Ismail,** ^**a**^ **Micaela Rayne Geborkoff,** ^**bc**^ **Thomas Werner** ^**bc**^ **and Marina Tanasova** ^*ab^

^**a**^ **Department of Chemistry, Michigan Technological University, 1400 Townsend Dr, Houghton, MI 49931, USA E-mail: mtanasov@mtu.edu**

^**b**^ **Health Research Institute, Michigan Technological University, 1400 Townsend Dr, Houghton, MI 49931, USA**

^**c**^ **Department of Biological Sciences, Michigan Technological University, 1400 Townsend Dr, Houghton, MI 49931, USA**

The authors apologize for this oversight. This correction does not affect the results or conclusions of the original article.

The Royal Society of Chemistry apologises for these errors and any consequent inconvenience to authors and readers.

